# Nucleocapsid Protein as Early Diagnostic Marker for SARS

**DOI:** 10.3201/eid1011.040516

**Published:** 2004-11

**Authors:** Xiao-Yan Che, Wei Hao, Yadi Wang, Biao Di, Kai Yin, Yin-Chao Xu, Chang-Sen Feng, Zhuo-Yue Wan, Vincent C.C. Cheng, Kwok-Yung Yuen

**Affiliations:** * First Military Medical University, Guangzhou, People's Republic of China;; † Center for Disease Control and Prevention of Guangzhou, Guangzhou, People's Republic of China;; ‡ Center for Disease Control and Prevention of Guangdong Province, Guangzhou, People's Republic of China;; § The University of Hong Kong, Hong Kong Special Administrative Region, People's Republic of China

**Keywords:** SARS-CoV, nucleocapsid protein, monoclonal antibody, enzyme-linked immunosorbent assay, early diagnosis, severe acute respiratory syndrome, dispatch

## Abstract

Serum samples from 317 patients with patients with severe acute respiratory syndrome (SARS) were tested for the nucleocapsid (N) protein of SARS-associated coronavirus, with sensitivities of 94% and 78% for the first 5 days and 6–10 days after onset, respectively. The specificity was 99.9%. N protein can be used as an early diagnostic maker for SARS.

Early laboratory diagnosis of the severe acute respiratory syndrome–associated coronavirus (SARS-CoV) is one step in preventing recurrence of a global outbreak. The availability of the complete genomic sequence of SARS-CoV has facilitated the development of a variety of diagnostic tests for SARS (*1*). Reverse transcription–polymerase chain reaction (RT-PCR) has been used as a rapid diagnostic test in most of the research centers during the last epidemic (*2**–**6*). However, early diagnosis of SARS remains a problem for nonresearch laboratories with little experience in molecular testing. We have developed an antigen-capture enzyme-linked immunosorbent assay (ELISA) based on monoclonal antibodies against the nucleocapsid (N) protein of SARS-CoV (*7*), a predominant antigen produced in the infected cell-culture filtrate. High levels of circulating N protein can be detected in the serum samples of patients with SARS. We attempt to demonstrate the temporal profile of the N protein and antibodies in serum samples from a large cohort of patients with SARS during the acute and convalescent phases of the disease. Our findings suggest that detecting N protein in serum can be used as an early diagnostic marker for SARS.

## The Study

During the 2003 SARS epidemic in Guangzhou, 420 serum specimens were collected from 317 patients 1–90 days after the onset of symptoms. The condition of all patients was diagnosed according to the World Health Organization criteria and confirmed by seroconversion or a fourfold increase in antibody titer against SARS-CoV by means of immunofluorescent testing. The N protein–capture ELISA was performed (*7*). Briefly, 100 µL of serum was added to the wells of a microtiter plate coated with a mixture of three anti-N protein monoclonal antibodies, and the plates were incubated at 37°C for 60 min. After the plates were washed, 100 µL of anti-N rabbit antiserum was added to the wells, and the plates were incubated at 37°C for 60 min. The wells were washed again and incubated for 1 h at 37°C with 100 µL of peroxidase-conjugated goat anti-rabbit immunoglobulin G (IgG). After the plates were washed, 100 µL of tetramethylbenzidine solution was added to each well. The experiments involving the use of serum samples from patients with SARS were performed within the safety cabinet of a biosafety level 2 laboratory.

**Figure 1:**
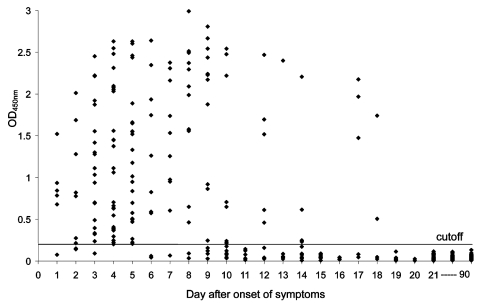
N protein detection in 420 serum samples from 317 patients with severe acute respiratory syndrome (SARS). Data represent the optical density at 450 nm (OD_450_) of undiluted serum samples. To establish the normal range of the N protein–capture enzyme-linked immunosorbent assay, serum specimens from 400 healthy blood donors were analyzed. The mean OD_450_ for these specimens, as determined by the assay, was 0.078, with a standard deviation of 0.023. The cutoff OD_450_ of the assay was then calculated as follows: cutoff = mean of OD_450_ from 400 normal serum + 5 x standard deviations = 0.19. Solid line represents cutoff value. The result was considered positive if a sample yielded OD_450_ above the cutoff.

**Figure 2:**
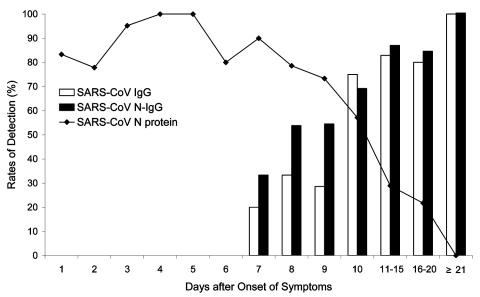
The profile of N protein detection in blood and antibody response to severe acute respiratory syndrome-associated coronavirus (SARS-CoV) from onset of symptoms to the convalescent phase. IgG, immunoglobulin G.

The results for the 420 serum specimens tested by the N protein–capture ELISA are shown in Figure 1. The N protein could be detected as early as day 1 and until day 18. In the 146 serum samples positive for N protein, the optical density (OD) value was highly variable from one sample to another on the same day. The sensitivity of detection was 94% (80 of 85 patients) with blood samples taken during the first 5 days and 78% (47 of 60 patients) for samples taken 6–10 days after onset of symptoms. The detection rate of N protein decreased to 27% on days 11–20 after onset of symptoms. Serum N protein was never detected beyond day 21. Using the same panel of the patient serum samples, we measured the N protein–specific IgG (SARS-CoV N-IgG) and SARS-CoV–specific IgG (SARS-CoV IgG) in serum samples by indirect ELISA, which progressively increased from day 7 onward (Figure 2). With the appearance of antibodies, the N protein detection rate decreased from day 10 after the onset of symptoms. However, from day 7 to day 18, a high level of N protein was still detectable in the serum samples from 11 patients with a mean of OD values of 1.65 when the SARS-CoV N-IgG had already increased to a level with a mean OD value of 1.18.

Serum samples from patients collected 4 years previously were used as negative controls. Patients thought to have cases of SARS on admission and later found to be uninfected by serologic testing, and healthcare workers who had close contact with SARS patients were tested for circulating N protein. Of the serum samples from non-SARS patients collected 4 years previously (n = 400), only one was weakly positive for N protein (OD = 0.288). We tested a total of 110 acute-phase serum samples from 105 patients, initially considered to have suspected SARS and later proven to be negative for SARS-CoV by serologic testing of convalescent-phase serum samples taken >28 days after onset of symptoms, and 315 serum samples from healthcare workers. All were negative for the N protein by capture ELISA. This finding resulted in a test specificity of 99.9% (1 of 825).

## Conclusions

Our results suggest that N protein in the serum samples of SARS patients can be detected as early as day 1 after disease onset. Although the level of circulating N protein was highly variable from one person to another from day 1 to day 18, development of SARS-CoV N-IgG appears not to be affected by N protein during the acute phase of the infection, 7 days after the onset of symptoms. The positive detection rate of N protein in serum samples within the first 10 days of infection is higher than that detected by RT-PCR (*8**,**9*). Furthermore, the variation in the reported sensitivity and specificity of RT-PCR may be related to the lack of standardization of the assay and the specimen collection (*6*). Therefore, this viral antigen-capture ELISA may have greater sensitivity, specificity, and ease and reliability of in-use performance than the nucleic acid amplification assay. Further comparative studies with nucleic acid amplification tests should be undertaken at clinical laboratories serving acute-care hospitals where rapid SARS diagnosis is vital.
